# Supplementary material for: Temporal trends of land-use favourability for the strongly declining little bustard: assessing the role of protected areas
Source: PeerJ. 2024 Jan 4;12:e16661. doi: 10.7717/peerj.16661 (PMC10771766; doi:10.7717/peerj.16661)
Supplement: Supplemental Information 2 — Little bustard abundance data shown in this table correspond to figures provided in official documents describing SPAs, not the data used in the analyses. [file peerj-12-16661-s002.docx]

| Nº | Name | Area (km^2^) | Province | Steppe area | Little bustard population | Percentage of extensive cereal |
| --- | --- | --- | --- | --- | --- | --- |
| 1 | La Nava-Campos Norte | 549.359 | Palencia  León  Valladolid | Yes | 40-50 males | 55 |
| 2 | La Nava-Campos Sur | 392.099 | Palencia  Valladolid | Yes | 6-8 males | 58 |
| 3 | La Nava-Rueda | 71.6703 | Valladolid | Yes | 5 males | 66 |
| 4 | Nature Reserve of Las Lagunas de Villafáfila | 325.49 | Zamora | Yes | 200-250 males | 90 |
| 5 | Llanuras del Guareña | 417.667 | Zamora | Yes | 75 males | 93 |
| 6 | Montes Aquilanos | 332.802 | León | No | - | 3 |
| 7 | Oteros-Campos | 316.853 | León  Valladolid | Yes | 110-165 males | 72 |
| 8 | Oteros-Cea | 44.4501 | León  Valladolid | Yes | 10-20 males | 79 |
| 9 | Páramo Leones | 69.1589 | León | Yes | 35-45 males | 23 |
| 10 | Penillanuras-Campos Norte | 132.418 | Valladolid  Zamora  León | Yes | 75-90 males | 50 |
| 11 | Penillanuras-Campos Sur | 238 | Zamora  Valladolid | Yes | 150-200 males | 50 |
| 12 | Riberas de Castronuño | 84.2108 | Valladolid | No | - | 35 |
| 13 | Tierra de Campiñas | 1394.45 | Valladolid  Ávila  Salamanca | Yes | 80-100 males | 43 |
| 14 | Tierra del Pan | 145.85 | Zamora  Valladolid | Yes | 60-80 males | 96 |
| 15 | Valdería - Jamuz | 97.132 | León | Yes | 240-260 males | 2 |
