# Supplementary material for: Temporal trends of land-use favourability for the strongly declining little bustard: assessing the role of protected areas
Source: PeerJ. 2024 Jan 4;12:e16661. doi: 10.7717/peerj.16661 (PMC10771766; doi:10.7717/peerj.16661)
Supplement: Supplemental Information 3 — “Horticultural”, “other cultures”, “trees”, and “water surface” were not included in the analyses due to their low representation in the study area. [file peerj-12-16661-s003.docx]

| Variable | Elements included in the variable | VIF values |
| --- | --- | --- |
| Artificial surface | Artificial, parks, paths and urban areas | 1.308 |
| Cereal | Barley (dry and irrigated), oats, rye, wheat (dry and irrigated), triticale, and other cereals (dry and irrigated) | 2.795 |
| Horticultural | Garlic, onions, strawberries, horticultural, potatoes, leeks, beets and carrots | --- |
| Legume | Alfalfa (dry and irrigated), chickpea, forage crops, green pea, vetch, bean, lentil, ervil and other leguminous | 1.935 |
| Oleaginous | Rapeseed, sunflower (dry and irrigated), and safflower | 1.530 |
| Other culture | Poppy, maize and other culture | --- |
| Seminatural | Fallow, wasteland, grassland, rocky area and bare ground | 1.768 |
| Trees | All tree species detected | --- |
| Water surface | Water | --- |
| Woody area | Abandoned woody crops, scrubs, aromatic plants (including lavender) and vineyards | 1.836 |
| Shannon index | Computed from the percentages of previous land covers unified | 1.555 |
