# Supplementary material for: Temporal trends of land-use favourability for the strongly declining little bustard: assessing the role of protected areas
Source: PeerJ. 2024 Jan 4;12:e16661. doi: 10.7717/peerj.16661 (PMC10771766; doi:10.7717/peerj.16661)
Supplement: Supplemental Information 4 — Both models include the standardized percentage of cereal, legumes and seminatural areas inside a 250 m radius around the census point. The geographical component was calculated including the standardised coordinates in the Legendre polynomial and selecting the variables backwards. The conspecific attraction was calculated as the number of males in 1.7 km radius around the census point. All degrees of freedom are 1. The R2 was 0.265, and 0.199 respectively; and the AUC was 0.867 and 0.820 respectively. [file peerj-12-16661-s004.doc]

| Model | Response variable | Explanatory variable | Estimate | χ2 | P |
| --- | --- | --- | --- | --- | --- |
| 1 | Presence of males | Seminatural areas | 0.5 | 2.875 | 0.090 |
|  |  | Cereal | 0.711 | 4.256 | 0.039 |
|  |  | Legume | 0.404 | 1.776 | 0.183 |
|  |  | Geographical component | 0.933 | 29.570 | <0.001 |
| 2 | Presence of males | Seminatural areas | 1.208 | 15.727 | <0.001 |
|  |  | Cereal | 1.478 | 17.783 | <0.001 |
|  |  | Legume | 0.798 | 6.550 | 0.011 |
|  |  | Conspecific attraction | 0.263 | 17.282 | <0.001 |
