# Supplementary material for: Temporal trends of land-use favourability for the strongly declining little bustard: assessing the role of protected areas
Source: PeerJ. 2024 Jan 4;12:e16661. doi: 10.7717/peerj.16661 (PMC10771766; doi:10.7717/peerj.16661)
Supplement: Supplemental Information 5 — The explanatory variables are the favourability value in the census point, the amount of neighbouring males measured as the number of males in 1.7 km radius, and the year when the census was carried out (standardized prior to the analyses). All degrees of freedom are 1, the R 2 for the model was 0.13 and the dispersion value was 0.412. χ2 represents the significance of likelihood ratio chi-squared statistics for each value. Sample size was 72 census points per year. [file peerj-12-16661-s005.docx]

| Response variable | Explanatory variable | χ2 | P |
| --- | --- | --- | --- |
| Number of females | Favourability | 1.381e6 | <0.001 |
|  | Males | 7.361e4 | <0.001 |
|  | Neighbouring males | 0.577 | 0.448 |
|  | Year | 1.576e4 | <0.001 |
