# Supplementary figures and images for: Temporal trends of land-use favourability for the strongly declining little bustard: assessing the role of protected areas
Source: PeerJ. 2024 Jan 4;12:e16661. doi: 10.7717/peerj.16661 (PMC10771766; doi:10.7717/peerj.16661)

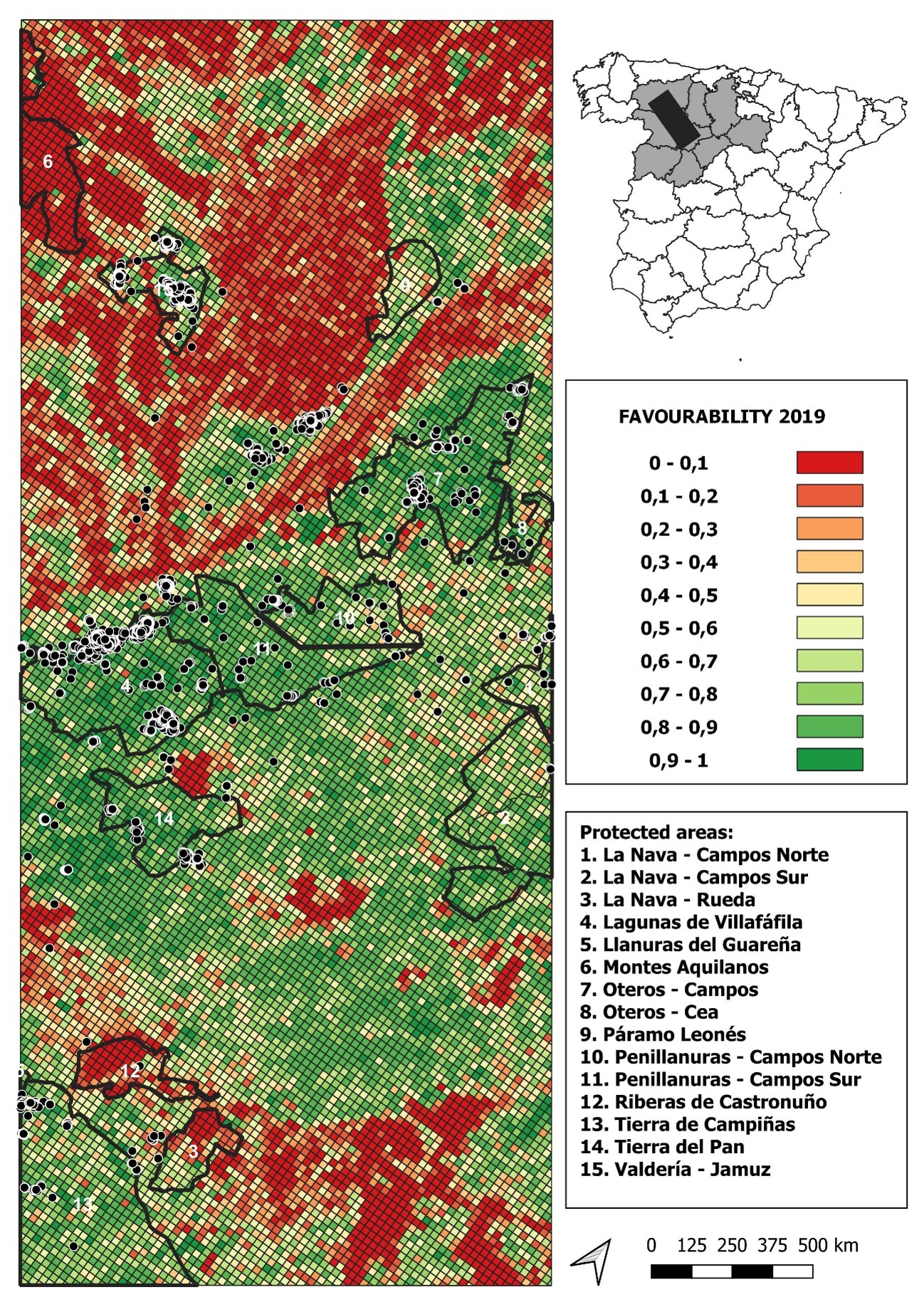

Supplement: Supplemental Information 6 [file peerj-12-16661-s006.jpg]

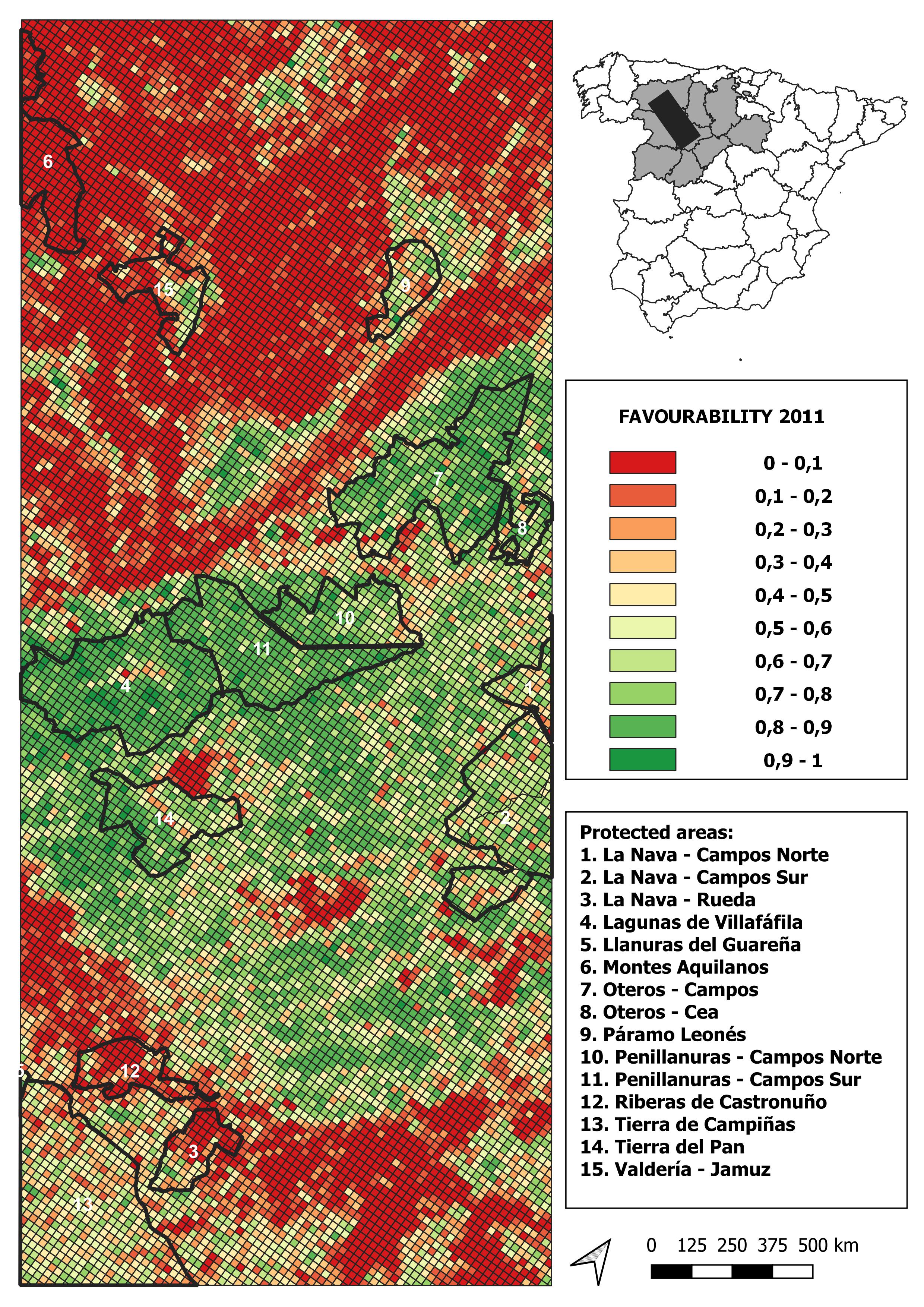

Supplement: Supplemental Information 7 [file peerj-12-16661-s007.jpg]

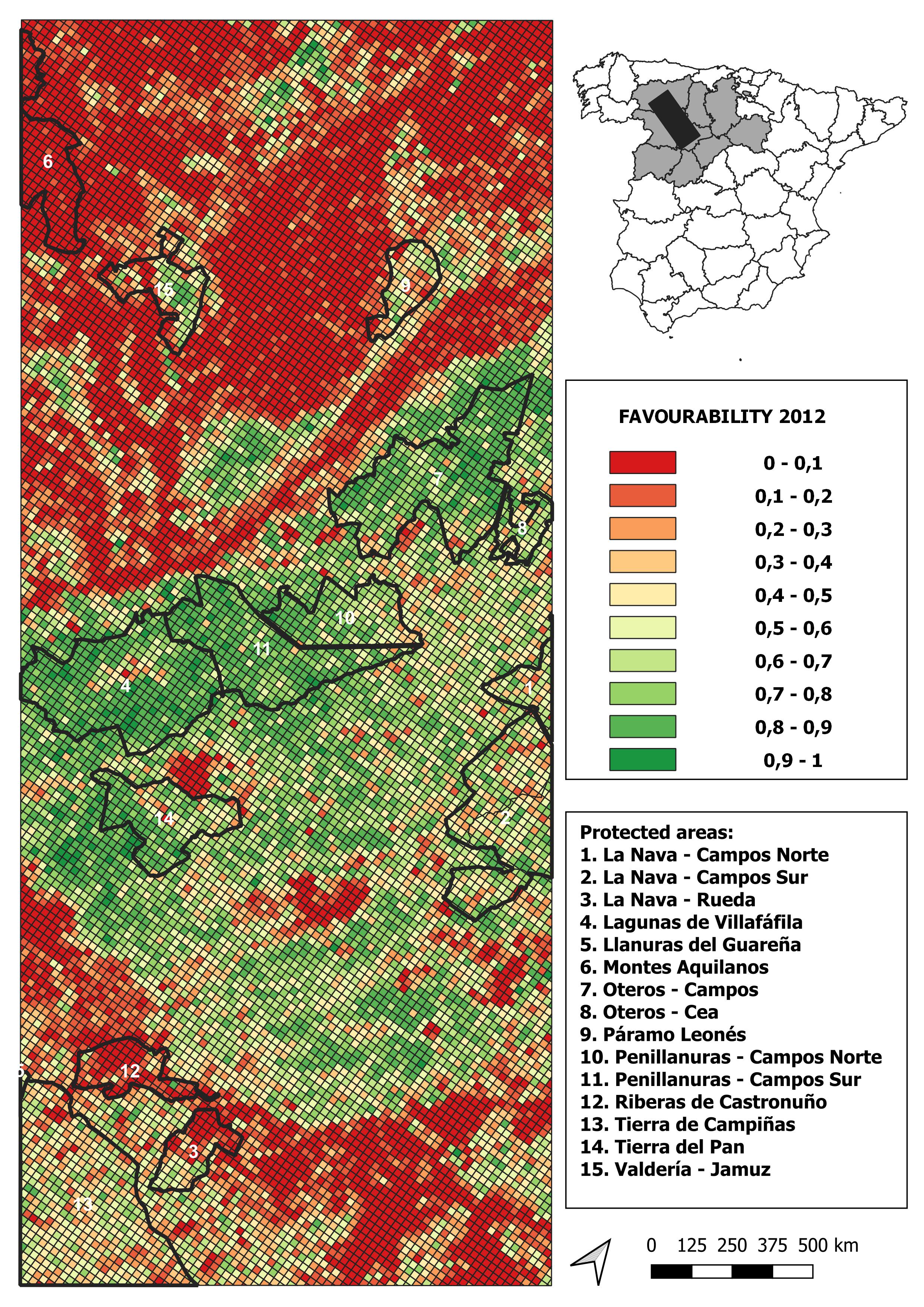

Supplement: Supplemental Information 8 [file peerj-12-16661-s008.jpg]

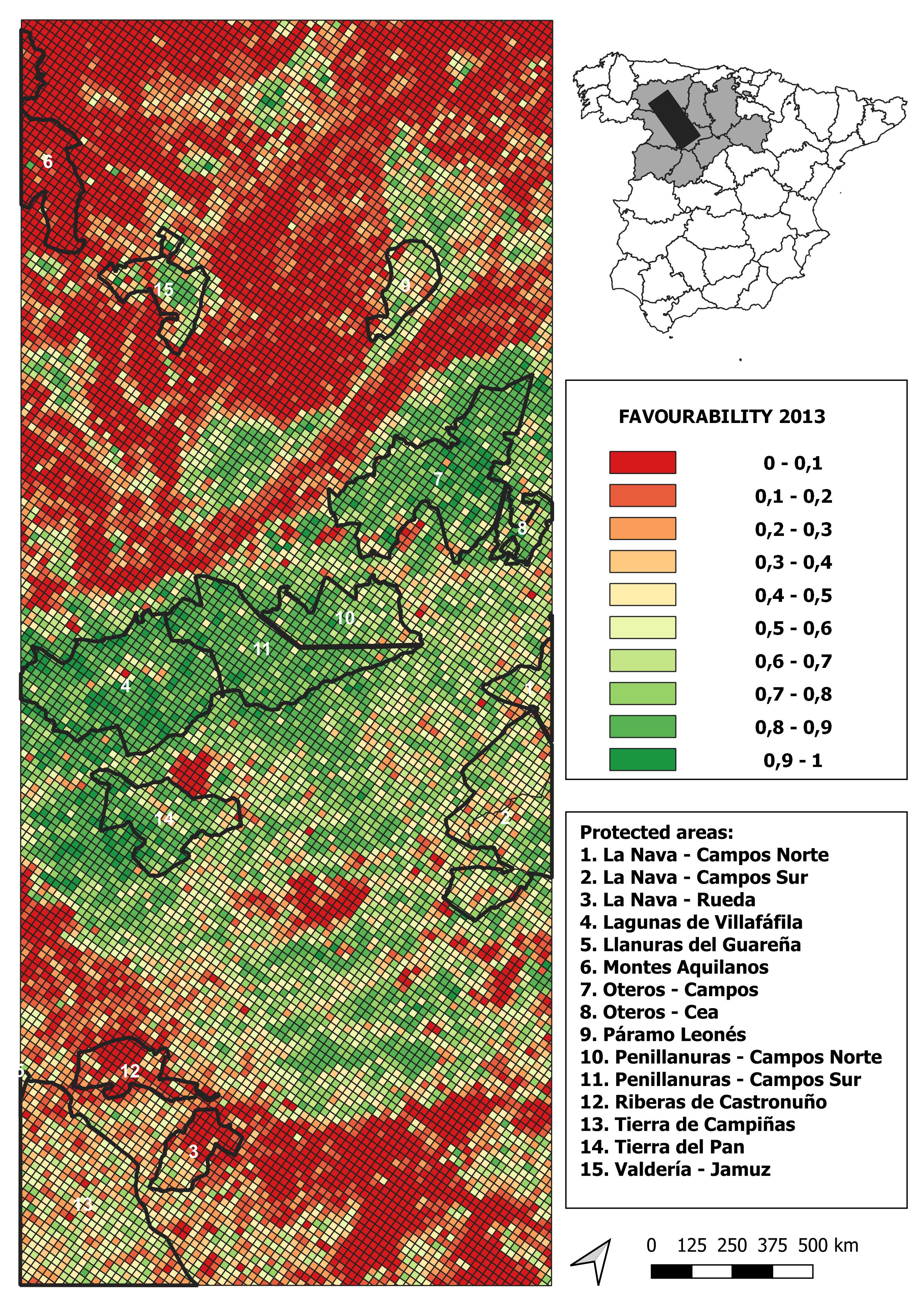

Supplement: Supplemental Information 9 [file peerj-12-16661-s009.jpg]

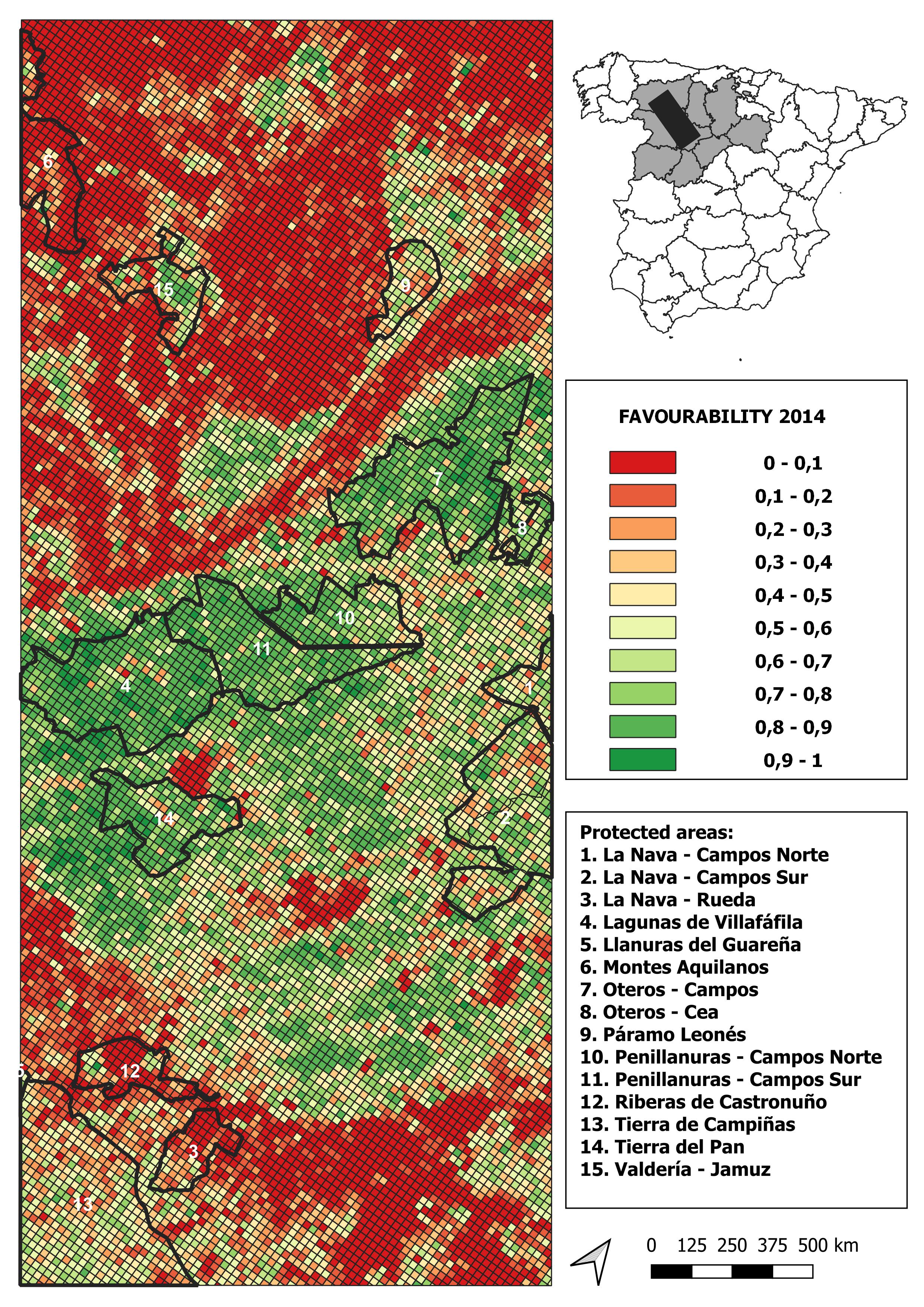

Supplement: Supplemental Information 10 [file peerj-12-16661-s010.jpg]

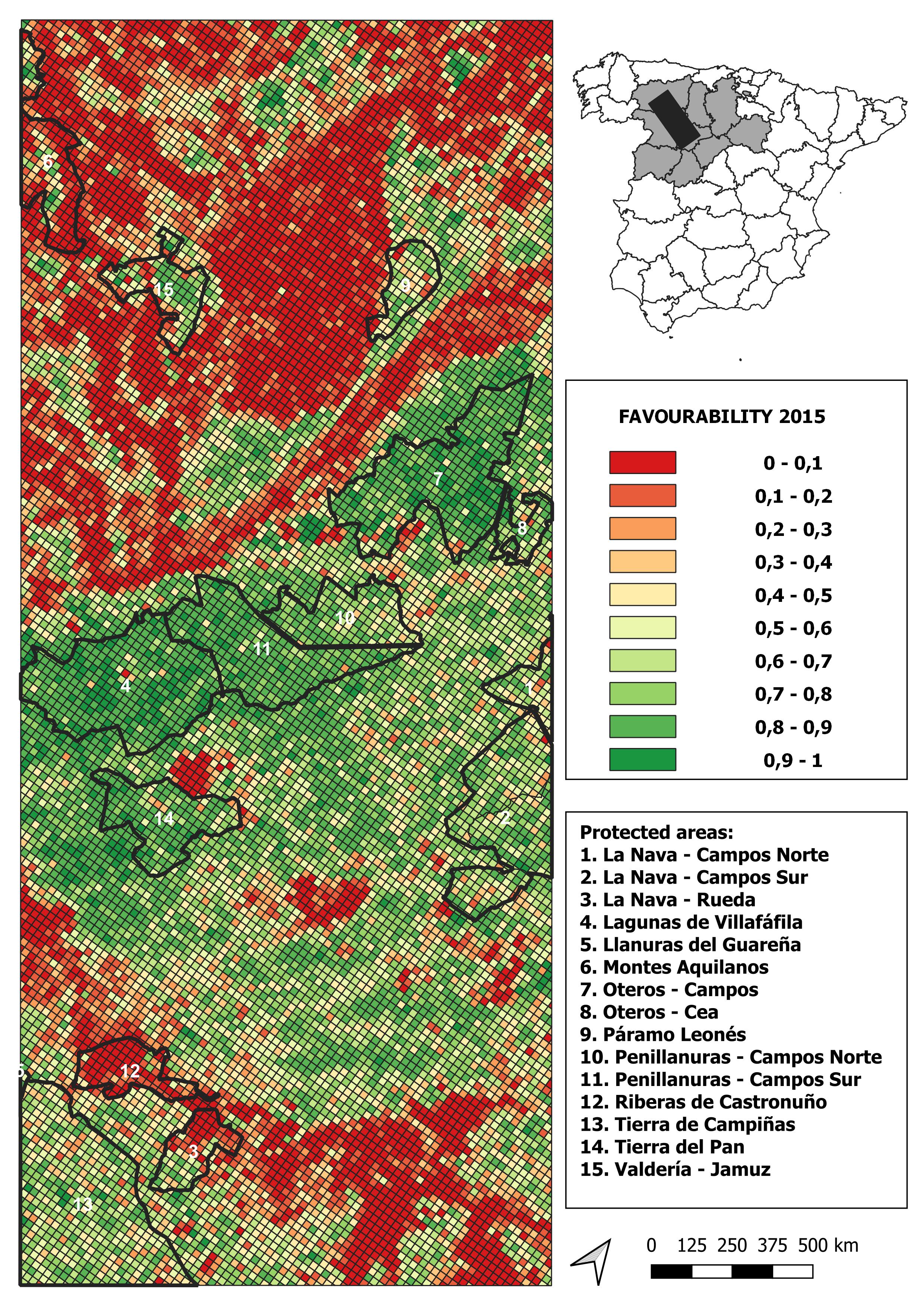

Supplement: Supplemental Information 11 [file peerj-12-16661-s011.jpg]

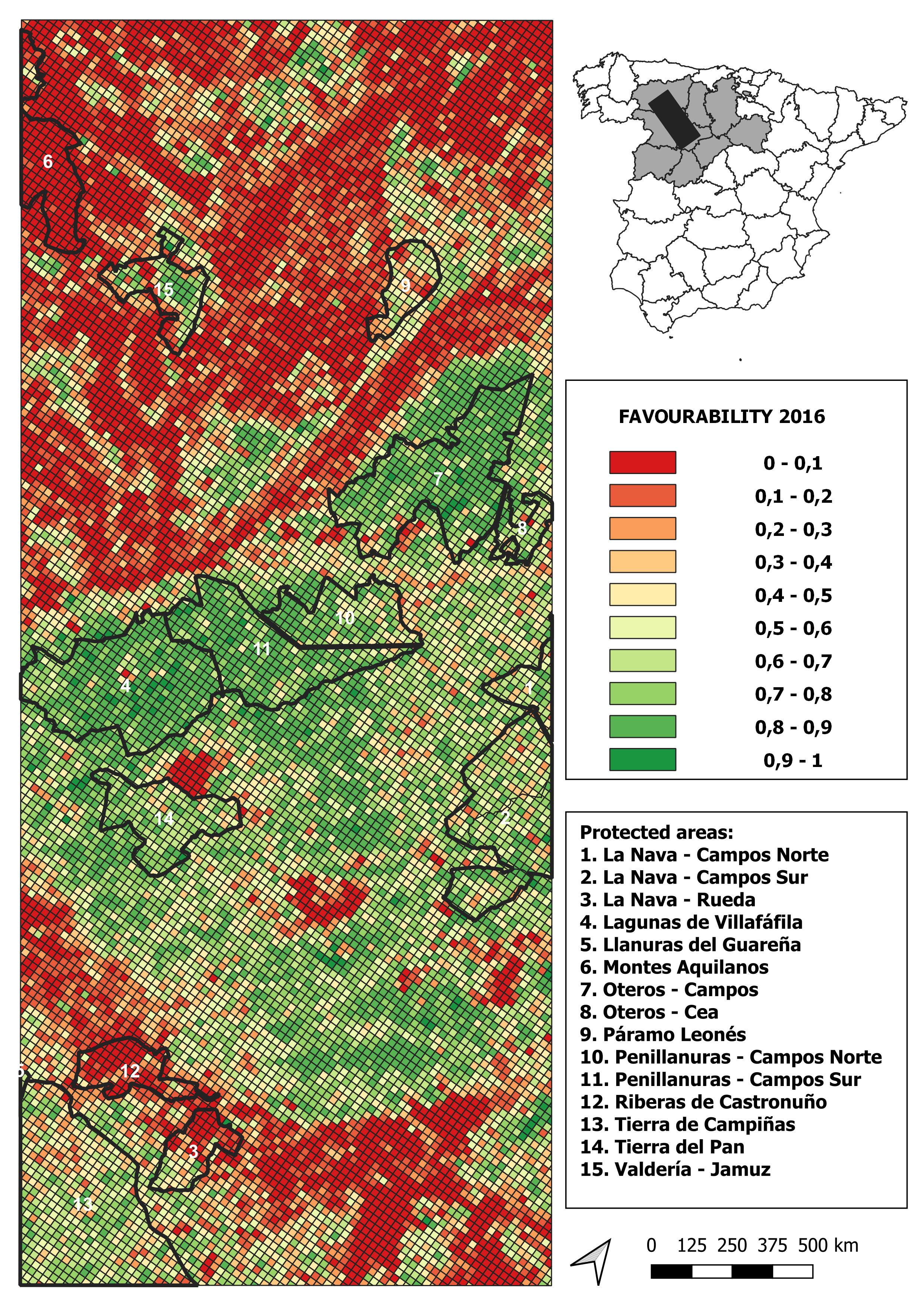

Supplement: Supplemental Information 12 [file peerj-12-16661-s012.jpg]

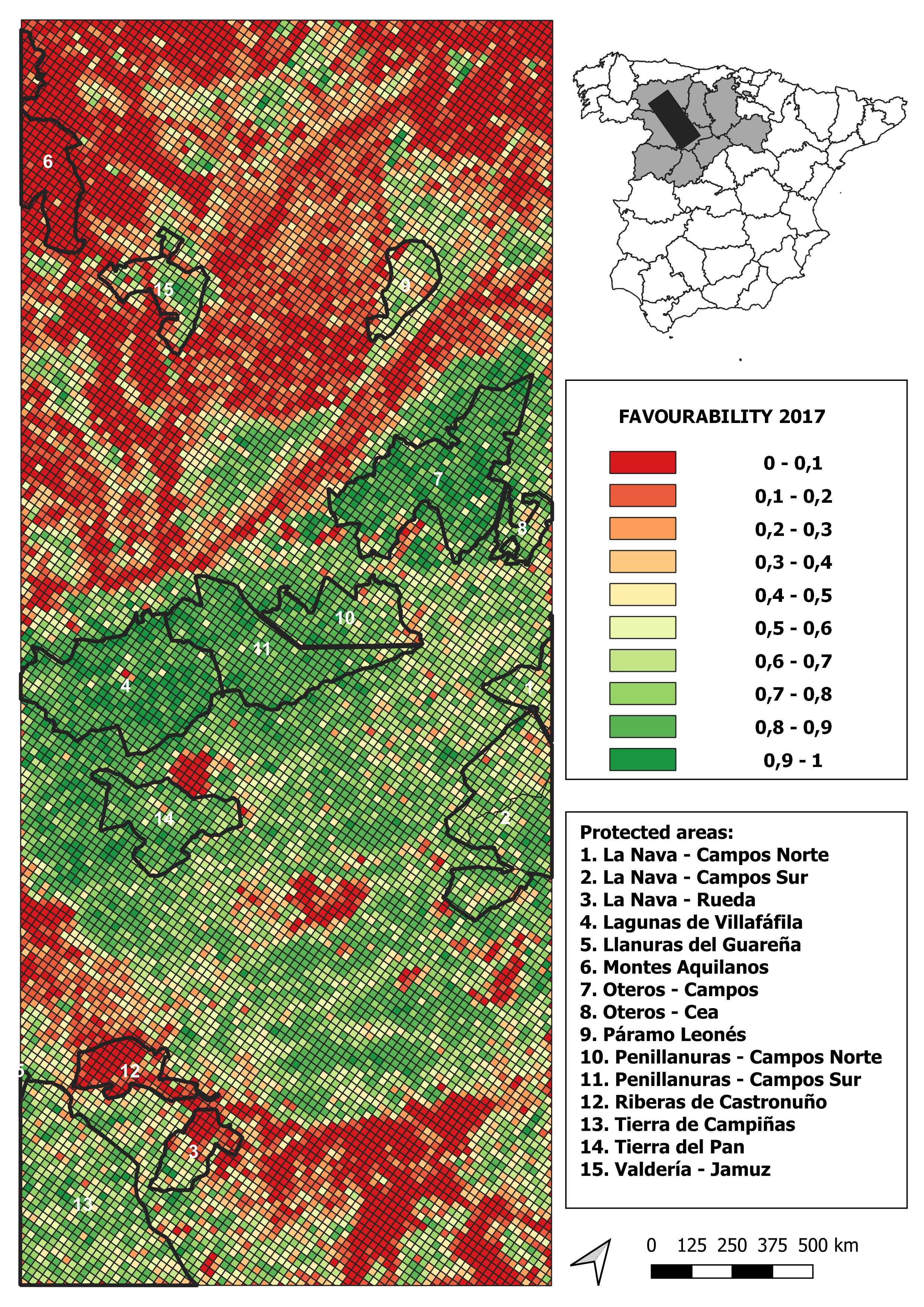

Supplement: Supplemental Information 13 [file peerj-12-16661-s013.jpg]

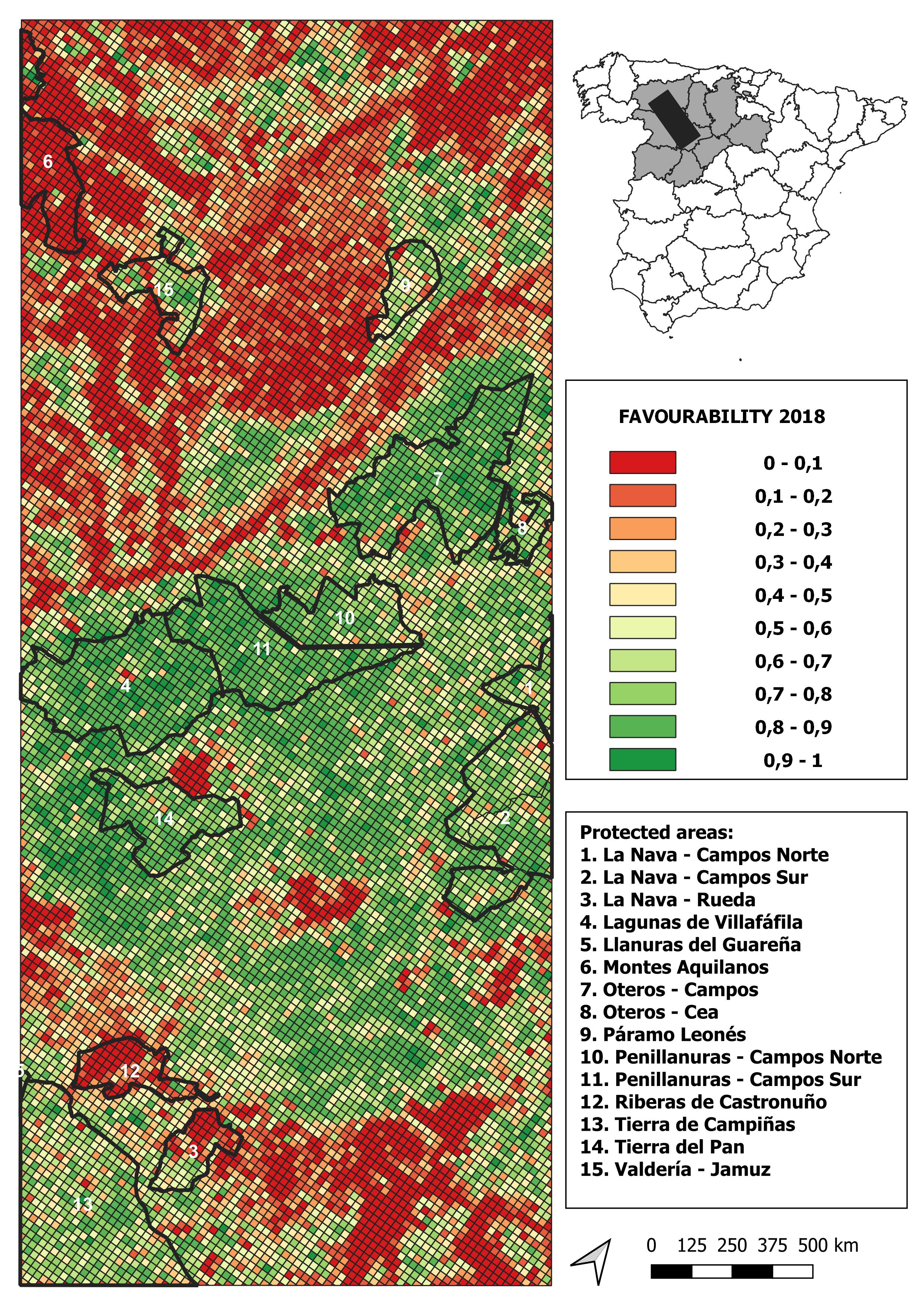

Supplement: Supplemental Information 14 [file peerj-12-16661-s014.jpg]

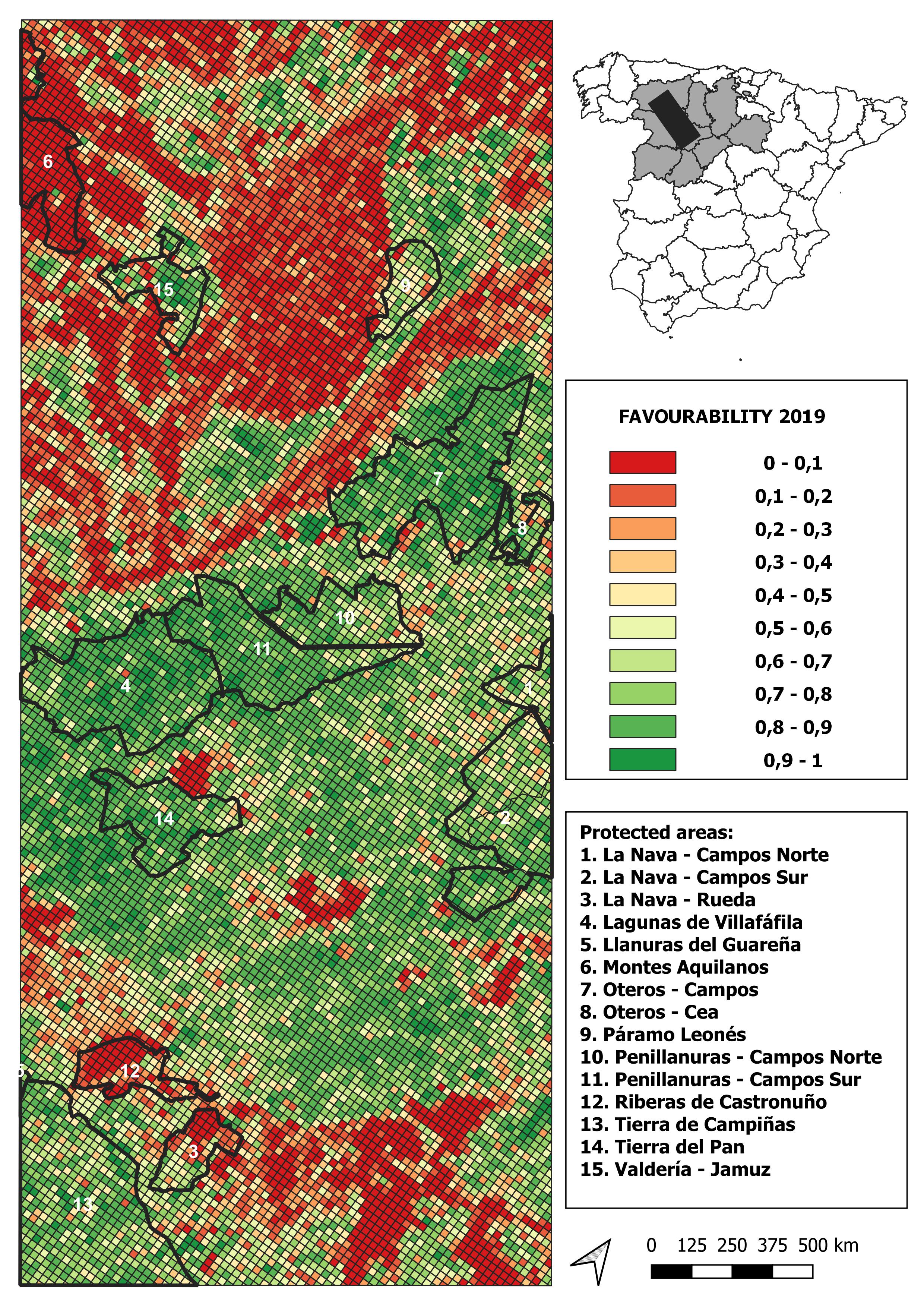

Supplement: Supplemental Information 15 [file peerj-12-16661-s015.jpg]

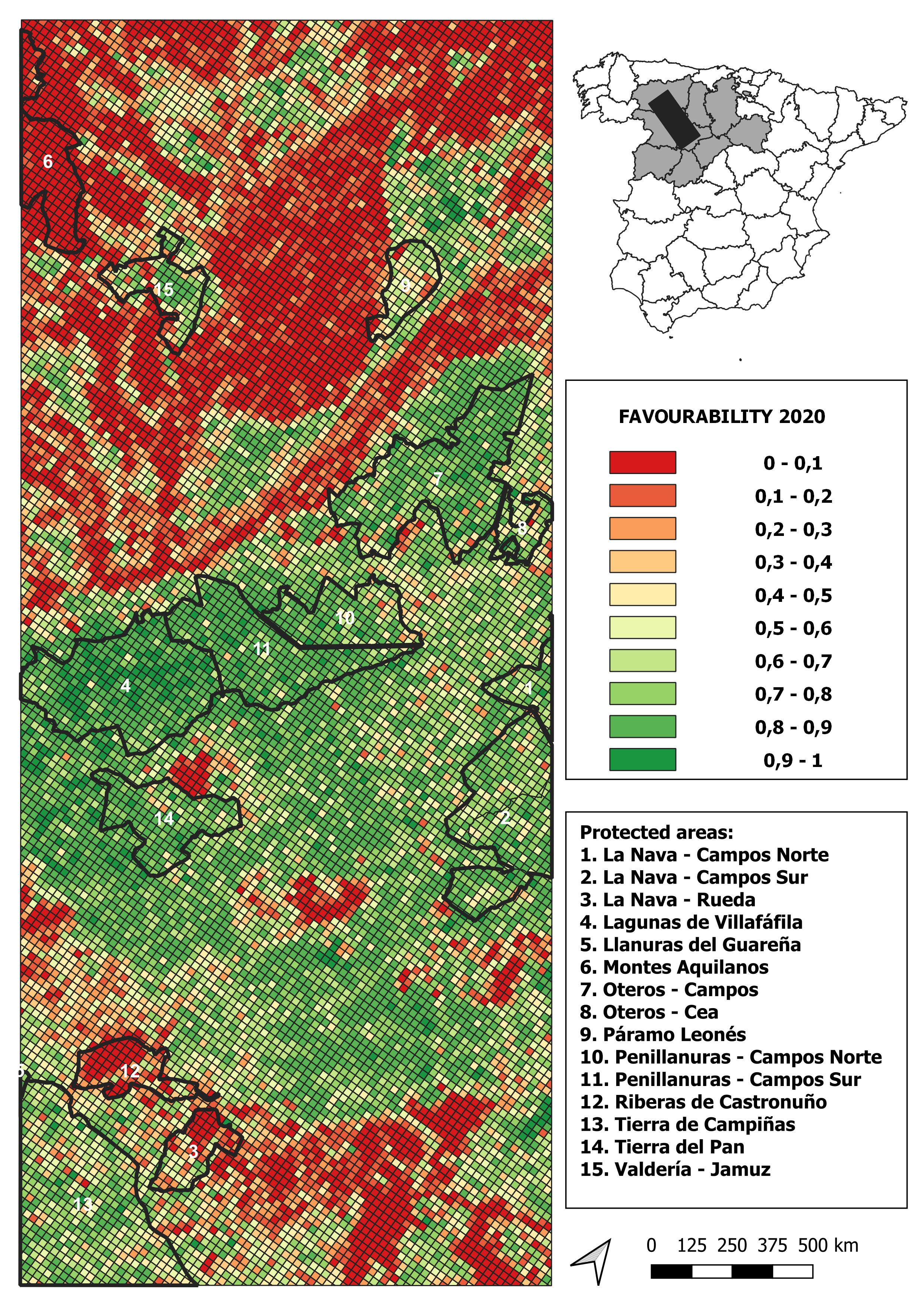

Supplement: Supplemental Information 16 [file peerj-12-16661-s016.jpg]

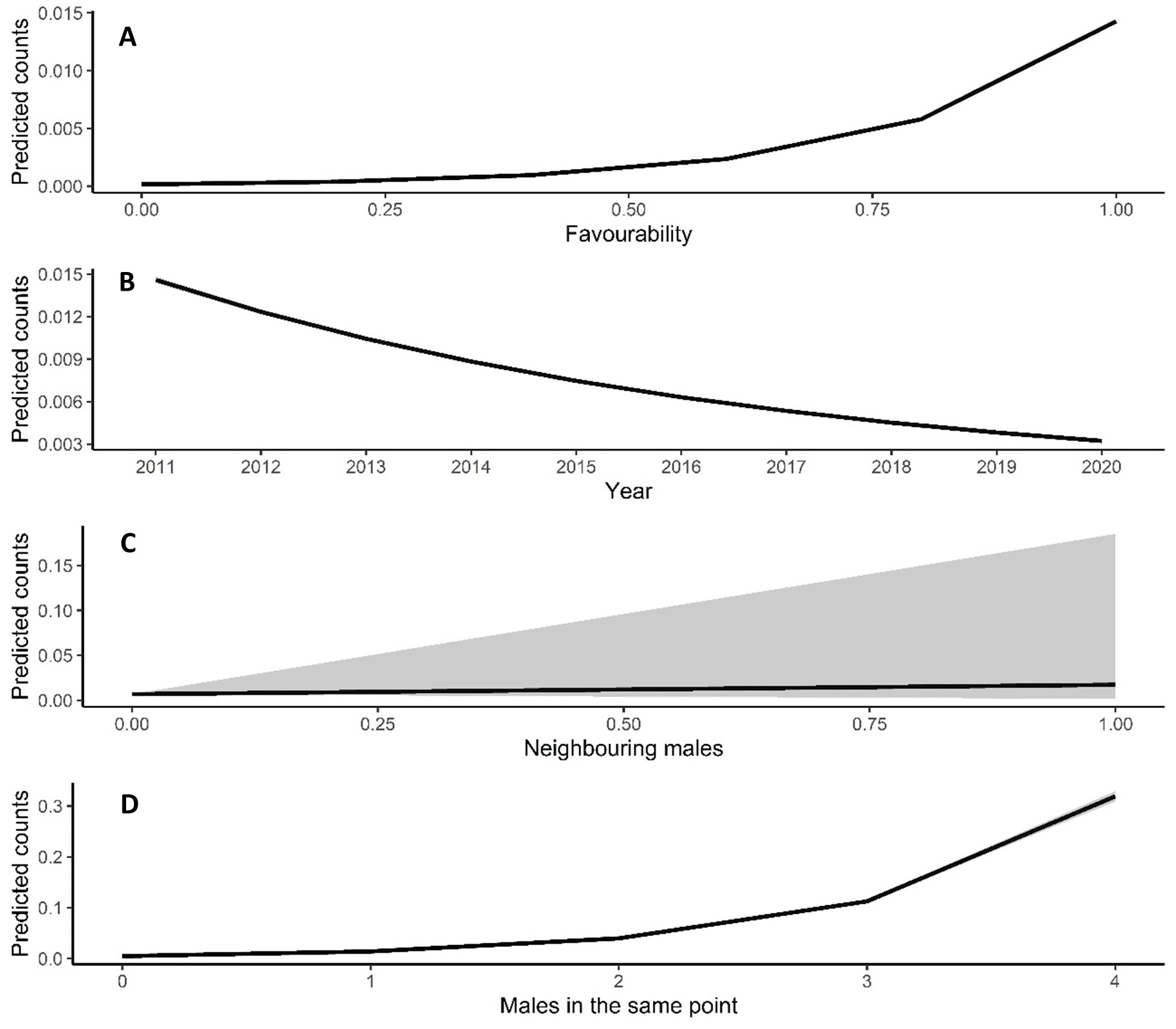

Supplement: Supplemental Information 17 — (A) shows the relationship of females predicted counts with favourability, (B) with year, (C) with the neighbouring males (i.e., the number of nearby males within a 1.7 km radius around each observation) and (D) with the number of males in the same census point. Mean values and 95% confidence intervals are shown. [file peerj-12-16661-s017.jpg]
